# Supplementary material for: A chromosome-scale assembly of the major African malaria vector Anopheles funestus
Source: Gigascience. 2019 Jun 3;8(6):giz063. doi: 10.1093/gigascience/giz063 (PMC6545970; doi:10.1093/gigascience/giz063)
Supplement: giz063_Response_to_Reviewer_Comments_Original_Submission [file giz063_response_to_reviewer_comments_original_submission.pdf]

Author's Response To Reviewer Comments

Close

Dear Editor,

We thank you and the anonymous reviewers for carefully reviewing our manuscript.

Before responding to the reviewers comments, we would like to note that between our original submission and this revision we became aware that the placement of the rDNA locus in our assembly is unusual compared to related anopheline species (i.e. it is placed on chromosome 3 rather than X). Due to its high copy number, the rDNA of any species is one of the most difficult to assemble regions and is rarely assembled correctly. Upon further inspection, the long-read and Hi-C data provided insufficient evidence to confidently place the rDNA locus onto an An. funestus chromosome. After consulting with the current users of the AfunF3 assembly (e.g. VectorBase), we decided not to alter the current version of the assembly so to not interrupt annotation and other ongoing analyses that depend on the AfunF3 coordinate system. We have added a brief description of this to the methods and the caption of Figure 3.

Please find below point by point response for reviewers' comments. Our responses are in blue.

Jay Ghurye, on behalf of all the co-authors

Reviewer reports:

Reviewer #1: The authors carried out a chromosomal level assembly of a major malaria vector, Anopheles funestus. The work itself is not technically novel because a much larger mosquito genome (Aedes aegypti) has been sequenced using similar techniques; however, the improved genome for this species will be certainly helpful for malaria research community. The authors have done an enormous amount of work with PacBio, 10x genomics, and HiC that has resulted in a chromosome level assembly. An. funestus is an important vector and the fragmented genome necessitated this assembly. It is also important to have more complete and contiguous genomes of multiple vectors instead of focusing only on An. gambiae.
The manuscript is very well written and is ready for publication. I recommend that this should be accepted for publication.

Authors' response: We thank you for the positive feedback on our work.

Reviewer #2: February 8, 2019

This manuscript entitled "a chromosome-scale assembly of the major African malaria vector Anopheles funestus" describes an updated reference genome for Anopheles funestus. This is a relatively understudied malaria vector and this work is an important step toward elucidating the genetics of this species. This resource was carefully assembled with cutting-edge sequencing methods and issues with redundancy were handled carefully, resulting in an excellent new resource for the field.

Authors' response: We thank you for the positive feedback on our work.

I have several minor comments and concerns:

1. Line 147: I am not familiar with QV scores. Consider adding percent value (e.g. 99.87%)

Authors' response: We have added the estimated percent accuracy of the assembly based on QV values to this line as well as to Table 1. New updated text reads: "The average Phred-scaled quality value of the new assembly was estimated as QV28 (99.84% identity) versus QV23 (99.49% identity) for the

Illumina-based AfunF1 assembly."

2. Line 150: This is surprising considering it is from 2 individuals from the same colony and there were no inversions detected. Do you think this could be an artifact from using 10X genomics reads for this? I think a bit more explanation is warranted for this finding.

We agree that this sentence does not have sufficient supporting evidence, so we have removed it. The end of this paragraph now reads "This independent data from a single mosquito of the FUMOZ colony indicates that the new AfunF3 assembly is of comparable accuracy to the prior Illumina-based assembly, and that the small differences between quality estimates could be due to genetic diversity within the colony."

3. Line 159: gap

Authors' response: Thank you for noticing this. We have fixed this typo.

4. Line 161: add italics to species name.

Authors' response: Thank you for noticing this. We have made it italics.

5. Line 165: Why would completeness be reduced? Do you think contigs were removed that only partially overlapped a larger contig?

Authors' response: We did not remove partially overlapping small contigs from the primary set. There are two reasons for reduced completeness. First, any contig with low coverage (< 40X) not overlapping with any other contigs is removed from the primary set. If these contigs had any of the core genes, they won't be included in the primary contig set. Second, if a BUSCO core gene is observed to be duplicated in multiple contigs, then we just keep one contig that has highest alignment score for that gene. This ends up in reducing completeness of core single copy genes because other possibly present BUSCO genes in the discarded contigs will be absent in the final primary contig set.

6. Line 171: specify contigs and primary AfunF3 assemblies.

Authors' response: We have made it clear in the text that we refer to both primary and complete AfunF3 assemblies.

Close
