# Supplementary material for: A chromosome-scale assembly of the major African malaria vector Anopheles funestus
Source: Gigascience. 2019 Jun 3;8(6):giz063. doi: 10.1093/gigascience/giz063 (PMC6545970; doi:10.1093/gigascience/giz063)
Supplement: giz063_Reviewer_2_Report_Original_Submission -- Brad Main — 2/8/2019 Reviewed [file giz063_reviewer_2_report_original_submission.pdf]

Reviewer Report

Title: A chromosome-scale assembly of the major African malaria vector Anopheles funestus

Version: Original Submission **Date:** 2/8/2019

Reviewer name: Brad Main

Reviewer Comments to Author:

February 8, 2019
This manuscript entitled "a chromosome-scale assembly of the major African malaria vector Anopheles funestus" describes an updated reference genome for Anopheles funestus. This is a relatively understudied malaria vector and this work is an important step toward elucidating the genetics of this species. This resource was carefully assembled with cutting-edge sequencing methods and issues with redundancy were handled carefully, resulting in an excellent new resource for the field.
I have several minor comments and concerns:
1. Line 147: I am not familiar with QV scores. Consider adding percent value (e.g. 99.87%)
2. Line 150: This is surprising considering it is from 2 individuals from the same colony and there were no inversions detected. Do you think this could be an artifact from using 10X genomics reads for this? I think a bit more explanation is warranted for this finding.
3. Line 159: gap
4. Line 161: add italics to species name.
5. Line 165: Why would completeness be reduced? Do you think contigs were removed that only partially overlapped a larger contig?
6. Line 171: specify contigs and primary AfunF3 assemblies.

Level of Interest

Please indicate how interesting you found the manuscript: Choose an item.

Quality of Written English

Please indicate the quality of language in the manuscript: Choose an item.

Declaration of Competing Interests

Please complete a declaration of competing interests, considering the following questions:

- Have you in the past five years received reimbursements, fees, funding, or salary from an organisation that may in any way gain or lose financially from the publication of this manuscript, either now or in the future?
- Do you hold any stocks or shares in an organisation that may in any way gain or lose financially from the publication of this manuscript, either now or in the future?

- Do you hold or are you currently applying for any patents relating to the content of the manuscript?
- Have you received reimbursements, fees, funding, or salary from an organization that holds or has applied for patents relating to the content of the manuscript?
- Do you have any other financial competing interests?
- Do you have any non-financial competing interests in relation to this paper?

If you can answer no to all of the above, write 'I declare that I have no competing interests' below. If your reply is yes to any, please give details below.

I declare that I have no competing interests

I agree to the open peer review policy of the journal. I understand that my name will be included on my report to the authors and, if the manuscript is accepted for publication, my named report including any attachments I upload will be posted on the website along with the authors' responses. I agree for my report to be made available under an Open Access Creative Commons CC-BY license (http://creativecommons.org/licenses/by/4.0/). I understand that any comments which I do not wish to be included in my named report can be included as confidential comments to the editors, which will not be published.

I agree to the open peer review policy of the journal

To further support our reviewers, we have joined with Publons, where you can gain additional credit to further highlight your hard work (see: https://publons.com/journal/530/gigascience). On publication of this paper, your review will be automatically added to Publons, you can then choose whether or not to claim your Publons credit. I understand this statement.

Yes Choose an item.
